# BariSurg trial: Sleeve gastrectomy versus Roux-en-Y gastric bypass in obese patients with BMI 35–60 kg/m^2^ – a multi-centre randomized patient and observer blind non-inferiority trial

**DOI:** 10.1186/s12893-015-0072-7

**Published:** 2015-07-18

**Authors:** Lars Fischer, Anna-Laura Wekerle, Thomas Bruckner, Inga Wegener, Markus K. Diener, Moritz V. Frankenberg, Daniel Gärtner, Michael R. Schön, Matthias C. Raggi, Emre Tanay, Rainer Brydniak, Norbert Runkel, Corinna Attenberger, Min-Seop Son, Andreas Türler, Rudolf Weiner, Markus W. Büchler, Beat P. Müller-Stich

**Affiliations:** Department of General, Visceral and Transplantation Surgery, University of Heidelberg, Im Neuenheimer Feld 110, 69120 Heidelberg, Germany; Institute of Medical Biometry and Informatics, University of Heidelberg, Im Neuenheimer Feld 305, 69120 Heidelberg, Germany; Study Centre of the German Surgical Society (SDGC), University of Heidelberg, Im Neuenheimer Feld 110, 69120 Heidelberg, Germany; Salem Hospital, Zeppelinstraße 11 – 33, 69121 Heidelberg, Germany; Department of General and Visceral Surgery, Städtisches Krankenhaus Karlsruhe, Moltkestraße 90, 76133 Karlsruhe, Germany; Department of General and Visceral Surgery, Agaplesion Bethesda Krankenhaus Stuttgart, Hohenheimer Straße 21, 70184 Stuttgart, Germany; Department of General and Visceral Surgery, Schwarzwald- Baar Klinikum, Klinikstraße 11, 78052 Villingen-Schwenningen, Germany; Department of Surgery, Caritas-Krankenhaus St. Josef, Landshuter Straße 65, 93053 Regensburg, Germany; Department of General and Visceral Surgery, Johanniter Krankenhaus, Johanniter GmbH, Johanniterstraße 3, 53113 Bonn, Germany; Department of Bariatric Surgery and Metabolic Surgery, Sana Klinikum Offenbach GmbH, Starkenburgring 66, 63069 Offenbach, Germany

**Keywords:** Sleeve gastrectomy, Roux-en-Ygastric bypass, Randomized controlled trial, Patient and observer blind trial, Long-term excess weight loss, Obesity related co-morbidity, Gastroesophageal reflux disease, Quality of life, Morbidity, Mortality

## Abstract

**Background:**

Roux-en-Ygastric bypass (RYGB) and sleeve gastrectomy (SG) rank among the most frequently applied bariatric procedures worldwide due to their positive risk/benefit correlation. A systematic review revealed a similar excess weight loss (EWL) 2 years postoperatively between SG and RYGB. However, there is a lack of randomized controlled multi-centre trials comparing SG and RYGB, not only concerning EWL, but also in terms of remission of obesity-related co-morbidities, gastroesophageal reflux disease (GERD) and quality of life (QoL) in the mid- and long-term.

**Methods:**

The BariSurg trial was designed as a multi-centre, randomized controlled patient and observer blind trial. The trial protocol was approved by the corresponding ethics committees of the centres. To demonstrate EWL non-inferiority of SG compared to RYGB, power calculation was performed according to a non-inferiority study design. Morbidity, mortality, remission of obesity-related co-morbidities, GERD course and QoL are major secondary endpoints. 248 patients between 18 and 70 years, with a body mass index (BMI) between 35–60 kg/m^2^ and indication for bariatric surgery according to the most recent German S3-guidelines will be randomized. The primary and secondary endpoints will be assessed prior to surgery and afterwards at discharge and at the time points 3–6, 12, 24, 36, 48 and 60 months postoperatively.

**Discussion:**

With its five year follow-up, the BariSurg-trial will provide further evidence based data concerning the impact of SG and RYGB on EWL, remission of obesity-related co-morbidities, the course of GERD and QoL.

**Trial registration:**

The trial protocol has been registered in the German Clinical Trials Register DRKS00004766.

## Background

### Rationale of the trial

The effect of bariatric surgery on obesity and related co-morbidities such as type 2 diabetes mellitus (T2DM) or hypertension is no longer doubted [1–3]. Reliable data showing improved overall patient survival and a reduced cancer incidence, especially in females, following bariatric surgery are available [4]. Additionally, bariatric surgery can now be performed safely and with acceptable morbidity and mortality [5, 6]. The procedures performed in the field of bariatric surgery are still evolving and include rather simple procedures such as gastric banding and more advanced techniques, such as biliopancreatic diversion or ileal transposition. Looking from a more general point of view, Roux-en-Y gastric bypass (RYGB) and sleeve gastrectomy (SG) are the most commonly performed procedures worldwide and in Germany, a finding that is likely due to their positive risk/benefit correlation [6]. However, RYGB is still considered superior to SG [7, 8]. This belief, however, is mostly based on historical considerations, as RYGB was one of the first bariatric procedures ever performed [9]. Because of recent evidence including systematic reviews and randomized controlled trials, SG has become more and more accepted as a stand-alone bariatric surgery procedure [9–17]. The systematic review data revealed, among other things, that excess weight loss (EWL) after SG was not significantly different from EWL following RYGB, 24 months after surgery [13]. This finding is consistent with that of Peterli et al., who also observed no significant differences in 12 month post-op EWL between SG and RYGB [9]. Another randomized controlled trial (RCT) from Finland, comparing SG and RYGB, revealed a significantly reduced operative time and complication rates in favour of the SG group [12]. As a result, the German statistic on obesity surgery for 2011 revealed for the first time, that more SG resections than RYBGs were performed [13, 18, 19]. Nevertheless, SG is still regarded with some scepticism due to the lack of valid long-term results and RCTs. Major criticism includes not only the effect of SG on EWL, but also on the course of obesity-related co-morbidities, on gastroesophageal reflux disease (GERD), and on quality of life (QoL) [20–23]. Furthermore, it seems that SG is correlated with rather specific complications such as fistulas and/or stenosis. The incidence of these complications is reported with the range 0 % to 17.5 % [19]. However, SG has a rather fast learning curve and can be performed within a short operative time. In addition, SG is considered less technically challenging than RYGB, due to the lack of anastomosis. Based on these unresolved controversies, further RCTs comparing SG and RYGB are urgently needed. The available RCT’s show that the clinical efficacy regarding EWL and T2DM remission between both procedures are comparable [14, 15, 24]. This goes along with similar morbidity rates even though it seems that the morbidity rate of RYGB in these RCTs is higher [12, 15].

**Table 1:** Inclusion and exclusion criteria

Inclusion criteria	Exclusion criteria
BMI 40-60 kg/m^2^	Lack of informed consent
BMI 35–40 kg/m^2^ with at least one obesity-related co-morbidity	Expected lack of compliance
Age 18–70 years	Previous bariatric surgery
	Pregnancy

### Objective

The primary endpoint of the BariSurg RCT, to compare EWL rates 2 years after SG and RYGB, was chosen based upon systematic review findings [13]. BariSurg will be a multi-centre, randomized controlled, patient and observer blind trial. Major secondary endpoints will be morbidity, mortality, re-operation rate, remission of obesity-related co-morbidities, the occurrence and course of GERD, QoL, the course of the dumping syndrome, and the EWL rate 60 months after surgery.

### Trial locations

The BariSurg RCT will be conducted at seven bariatric centres: the University of Heidelberg, Städtisches Klinikum Karlsruhe, Agaplesion Bethesda Krankenhaus Stuttgart, Schwarzwald-BaarKlinikum (Villingen-Schwenningen), Caritas-Krankenhaus St. Josef (Regensburg), Johanniter Krankenhaus (Bonn) and Sana Klinikum Offenbach GmbH (Offenbach).

## Methods/Design

### Trial design

The BariSurg trial is a multi-centre, randomized controlled, patient and observer blind trial.

### Sample size

A total of 248 patients will be randomized, with 124 patients assigned to each treatment arm. A dropout rate of approximately 20 % is considered realistic and has been accounted for the total number of randomized patients.

### Patient selection criteria

Patients between 18 and 70 years old with a BMI between 35 and 60 kg/m^2^ with indication for bariatric surgery according to the most recent German S3 guidelines will be eligible. Patients with a BMI of 35–40 kg/m^2^ need to have at least one obesity-related co-morbidities such as T2DM or hypertension (see Table 1).

### Recruitment and timelines

Patients will be recruited by the above mentioned seven participating centres. The recruitment period is estimated at 18 months. The time from enrollment of the first patient to study completion of the last, will be approximately 78 months, and the proposed duration of the entire trial is 90 months.

### Randomization

Patients fulfilling all inclusion without meeting any exclusion criteria and after receiving their informed consent, randomization will be performed intraoperatively via an internet-based randomization tool (http://www.randomizer.at). Randomization is performed by block randomization and will be stratified for each centre until the enrolment goal of 248 patients has been reached.

### Interventions

Patients will be asked to complete standard preoperative diagnostics including esophagogastroduodenoscopy (EGD), endocrine assessment and psychosomatics, and will also fill out four questionnaires (Short-Form-36 Health Survey (SF-36) [25], Gastrointestinal Quality of Life Index (GIQLI) [26], the Dumping questionnaire (Sigstad score) and Gastrointestinal Symptom Rating Score (GSRS) [27, 28]). Two weeks before surgery, patients will be asked to reduce their weight by following a low calorie liquid diet [29]. Preoperatively, a single-shot antibiotic prophylaxis will be given. For the surgical procedure, the patient will be placed in the supine position 45° (reverse Trendelenburg).

### Roux-en-Y-gastric bypass

After the gastroesophageal junction is identified, the stomach is transsected with a linear stapler 6 cm below the junction. A pouch with a 4–6 cm height and 14–16 mm width is created using a 42 French tube. A 70 cm biliopancreatic limb is defined and an end-to-side gastroenterostomy will be performed using either a 30 mm linear or 25 mm circular stapling technique. The common channel (side-to-side jejunojejunostomy) will be stapled after another 150 cm antecolic limb. To identify a leak of the proximal anastomosis methylene blue is applied through a nasogastric tube [30]. All patients will be discharged with the recommendation of oral intake of multivitamin tablets twice daily. During the follow up examination vitamin levels will be assessed regularly. In case of vitamin deficiency substitution of vitamins will be performed.

### Sleeve gastrectomy

The gastroepiploic vessels are divided 6 cm prepyloric along the great curvature, until the angle of His and the left crus of the diaphragm are visible. Along with a 42 French bougie, the stomach is then resected using linear stapler devices. At the angle of His the stapler line is sutured. The sleeve is checked for leakage using methylene blue. All patients will be discharged with the recommendation of oral intake of multivitamin tablets twice daily.

### Study visits

**Fig. 1:**
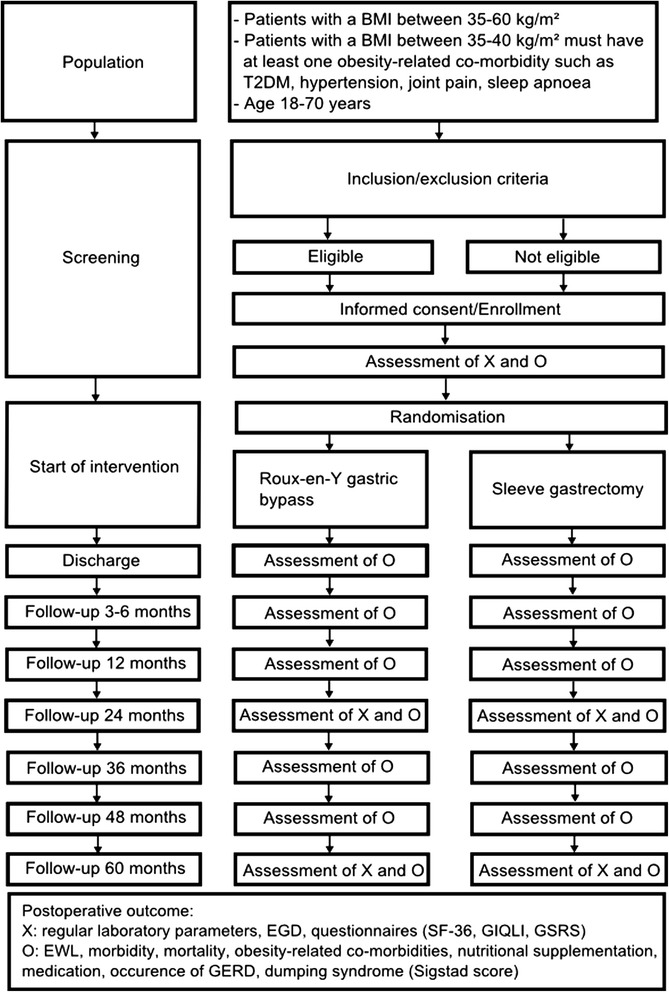
Flow chart of BariSurg trial, showing the timeline and the course for trial participants. Abbreviations: BMI: body mass index, T2DM: type 2 diabetes mellitus, EWL: excess weight loss, EGD: esophagogastroduodenoscopy, SF-36: Short-Form-36 Health Survey, GIQLI: gastrointestinal quality of life index, GSRS: gastrointestinal symptom rate score, EWL: excess weight loss, GERD: gastroesphageal reflux disease

Study documentation and visits of patients will be performed by both surgeons and study nurses. Since the trial is designed as observer and patient blind RCT, information about the surgical procedure will not be reported during the follow-up examinations. There will be 9 study visits during the BariSurg RCT (see Fig. 1). During the first postoperative year, study visits will be performed at discharge, 3–6 months and 12 months postoperatively. Then visits will be performed annually. Every postoperative study visit includes data collection of weight, morbidity, mortality, course of obesity-related co-morbidities (including laboratory parameters and current medication), analysis of nutritional supplementation, occurrence of GERD and analysis of dumping syndrome using the Sigstad score. In addition to the above mentioned information, extended study visits will yearly assess general laboratory parameters, EGD results and the questionnaires (SF-36, GIQLI, GSRS, see Fig. 1).

### Risk-benefit ratio

SG is associated with a morbidity rate between 0–17.5 % and a mortality rate between 0–1.2 % [13]. The most frequently occurring postoperative complications are insufficiencies/fistulas at the staple line, leakage, stenosis of the sleeve and dilatation of the sleeve [5]. RYGB has a morbidity rate of 2-10 % and a mortality of 0.5-0.8 % [7, 12, 15]. Frequently observed complications include anastomotic insufficiency, especially with regard to the gastrojejunostomy, and dumping syndrome. Dumping syndrome is observed in 42 % after RYGB and up to 29 % after SG [31–33]. Therefore patients will be assessed for dumping syndrome. If the diagnosis dumping syndrome is confirmed patients will get the optimal treatment for dumping syndrome [28]. For both bariatric procedures, nutritional supplementation might be necessary for the remainder of the patient’s life.

### Outcome

The summary of the primary and the secondary endpoints depicts different points of view concerning the efficacy and safety of bariatric procedures. In doing so, medical issues as well as patient assessment can be analyzed.

### Primary endpoint

The primary endpoint is defined as EWL at 24 months after surgery (RYGB or SG). Power calculation was made according to the systematic review of Fischer et al. [13].

### Secondary endpoints

Long-term EWL at 60 months after surgery, morbidity, mortality, course of obesity-related co-morbidities (T2DM, hypertension, joint pain, sleep apnoea, dyslipidemia), vitamin status, incidence and/or course of GERD and QoL (as defined by the SF 36) and dumping syndrome (as defined by the Sigstad score) will form the secondary endpoints. The course of obesity-related co-morbidities is assessed by laboratory examinations and current medication use. T2DM remission will be measured by HbA1c, fasting glucose and medication use. Hypertension will be monitored by medication and blood pressure measurements. The course of joint pain and dyslipidemia are assessed by pain and respectively lipid metabolism medication. The course of sleep apnoea is analysed by patients’ application of CPAP-mask. Nutritional substitution will be assessed using a questionnaire which was developed in the bariatric centre of the University of Heidelberg. GERD incidence and course of GERD will be assessed with EGD and by using GSRS and GIQLI as standardized questionnaires. To evaluate QoL, the validated SF-36 questionnaire will be used. The Dumping score will be evaluated by using the validated Sigstad score. Secondary endpoints will be evaluated preoperatively, at discharge, 3-6, 12, 24, 36, 48 and 60 months postoperatively. Questionnaires will be filled out 12, 24 and 60 months after surgery. The EGD will be performed 12 and 60 months postoperatively.

### Data management

Trial-relevant data will be documented in the case report form (CRF). The original CRFs will remain at the investigating centre and copies will be sent to the primary investigating centre, i.e. University Heidelberg. Data extraction and analysis will be made by the contract research organization, R&P Ryschlick and Partner GmBH (Burscheid, Germany). Data will be analyzed for completeness, validity and plausibility. The investigator will be consulted in case of uncertainty of the data.

### Safety evaluation and reporting of adverse events

Patients will receive regular medical treatment, including any necessary emergency treatment, throughout the duration of the trial. Serious adverse events (SAE) are defined as the need for re-operation, prolonged hospitalization, life-threatening situations, readmission for any reason, and death. The principal investigator must be informed about all occurring SAEs within 24 hours of knowledge of the event. All SAEs must be reported to the principle investigator.

### Unblinding

Blinding of patients has been done many times even in surgery [34–36]. Besides randomization, blinding of patients (and observers) reduces bias. Subsequently, internal and external validity will be increased. The fact that this study will be a patient and observer blinded study made a thoroughly ethical and clinical evaluation necessary. Based on the given evidence there is reliable data available that both procedures are similar in their clinical efficacy regarding excess weight loss, diabetes remission and complication rates [13–15]. Taking aside historical considerations and looking only at the RCT regarding RYGB and SG one can truly suggest to patients that both procedures are equally effective and safe.

During the BariSurg trial, all patients get extensive oral and written information about both procedures with all relevant pros and cons. Patients are informed that they will not know which procedure will be performed. Randomization will be performed intraoperatively after confirming that both procedures can be performed safely. All relevant documents including operation report and discharge letter state that the patient was enrolled in a randomized controlled patient and observer blind clinical trial comparing RYGB and SG and that the patient does not know about the procedure. These documents also include an emergency number which patients or physicians can call any time (24 hours, 365 days service) in case unblinding is necessary. Thus, blinding patients during the BariSurg trial is reasonable both from a clinical point of view as well as from ethical considerations.

### Statistical methods

#### Sample size

According to a systematic review by Fischer et al. the mean EWL two years after SG and RYGB was 56.1 % and 68.3 % respectively, with a common standard deviation of 22.5 % [13]. Based on these results, the null hypothesis is that there exits inferiority of SG compared to RYGB in EWL 24 months after surgery. Based on the assumption that a difference in EWL of 10 % or more is clinically relevant, the margin to consider EWL as similar is set to 9 %. The power calculation for a non-inferiority trial with a one-sided t-test (alpha = 0.025 % and beta = 20 %) and a standard deviation of 22.5 % revealed that 99 patients per intervention group need to be randomized. Since a high dropout rate of 20 % is expected, a further 25 patients per intervention group should be randomized. Hence a total of 124 patients for each group will be recruited (overall 248 patients).

### Analysis of primary endpoint

The primary endpoint is percent of EWL 24 months after surgery. To formalize the statistical approach, the following notations will be used: μRYGB/μSG, population mean of primary endpoint in RYGB/SG group. The following one-sided non-inferiority test problem is defined as

- H0: μRYGB - μSG > = delta vs.
- H1: μRYGB - μSG < delta

(For definition and specification of delta, see sample size calculation). This hypothesis will be tested using a one-sided t-test applied to each protocol population (see below). A low number of missing values for the primary endpoint is expected. If any values are missing, they will be replaced by methods of multiple imputation.

### Secondary analyses

Concerning secondary endpoints, exploratory data analysis will be performed and appropriate summary measures for the empirical distribution, as well as descriptive two-sided p-values, will be calculated. Homogeneity of the treatment groups will be described by comparing the baseline values. Each patient’s allocation to the different analysis populations [full analysis set (FAS) according to the intention-to-treat (ITT) principle, per protocol (PP) analysis set, safety analysis set] will be defined prior to analysis and documented in the analysis plan prior to database closure. During the data review, deviations from the protocol will be assessed as 'minor' or 'major'. Major deviations from the protocol will lead to the patient's exclusion from the PP analysis set. In addition to the evaluation of PP, an ITT analysis will be performed as a sensitivity analysis.

### Safety measures

Safety analysis includes frequency of SAEs and complications. Homogeneity of the study arms will be described by comparing demographic data with baseline values.

### Withdrawals and stopping guidelines

Patient withdrawal from the trial is possible at any time and without explanation. The trial will be ended in cases of insufficient patient recruitment and a high rate of SAEs due to SG or RYGB.

### Data safety monitoring board

Reports of SAEs will be collected by an independent data and safety monitoring board (DSMB). Furthermore, the DSMB will inform the trial management about relevant imbalances between the two groups.

### Trial organization and administration

#### Ethical considerations

SG and RYGB are frequently performed, standard techniques in bariatric surgery. The overall complication rate in SG and in RYGB described by Birkmeyer et al. is 5.9 % and 10.3 % respectively. A mortality of 0 % in SG and 0.3-0.9 % in RYGB was observed [12, 15, 37]. All participating centres perform both techniques frequently. In this manner morbidity and mortality rate can be expected like above mentioned. Furthermore, all participating centres received positive approvals of the according ethic committees (i.e. the University of Heidelberg from the Heidelberg ethics committee (S500/2012), Johanniter Hospital (Bonn) (2015179/2014), Städtisches Klinikum Karlsruhe, Schwarzwald-Baar-Klinikum (Villingen-Schwennigen), Agaplesion Bethesda Krankenhaus Stuttgart from the ethics committee of the State Board of Physicians of Baden-Württemberg which is responsible for the mentioned hospitals (B-F-2014-059)). The Caritas-Krankenhaus St. Josef (Regensburg) requested formal information of the ethics committee of the Bavarian State Board of Physicians to be legally considered a positive ethic vote.)

### Good clinical practice

The BariSurg trial will be conducted according to national and international trial standards (ICH-GCP, Declaration of Helsinki 2008).

### Registration

This trial is registered in the German Clinical Trials Register (DRKS00004766).

## Discussion

The positive effects of bariatric surgery on weight loss and obesity-related co-morbidities are no longer doubted. In addition, these procedures can also be performed safely with low mortality and morbidity [5, 12, 38]. The range of available bariatric procedures is tremendous [7, 17, 39–45]. Almost every year, a “new” procedure is focussed upon within the scientific community [18, 19]. However, there are only few RCTs comparing the two most commonly performed bariatric procedures, i.e. RYGB and SG with regard to actual weight loss and/or improvement of obesity-related co-morbidities in the mid- and long-term [12, 15]. It is therefore impossible to advocate any particular, bariatric surgical method, because one still does not know which patient benefits most from which procedure. A systematic review revealed that the EWL after 24 months is not statistically different between RYGB and SG [41]. In addition, the same publication demonstrated the poor data quality among publications dealing with SG. The urgent questions concerning comparisons between SG and the current gold standard of RYGB with respect to long-term EWL, course of obesity-related co-morbidities, course of GERD and QoL, are still not answered. In the last 2 years, however, a small number of RCTs were started with the goal of examining some of these issues [12, 14, 15, 17, 39]. To our knowledge, the BariSurg trial will be the first multi-centre, randomized controlled patient and observer blind clinical trial with a sufficient sample size analyzing hard clinical endpoints such as mid- and long-term EWL, morbidity and mortality. In addition BariSurg will also answer some of the urgent questions associated with SG, such as course of obesity-related co-morbidities, dumping syndrome and GERD. Thus, BariSurg will contribute to class 1B evidence, which enable future class 1A evidence in form of meta-analyses. The remission of obesity-related co-morbidities, such as T2DM, following bariatric procedures is already known [1–3, 40]. In particular, the RYGB has been considered as a potential therapy for T2DM, even in patients with a BMI of less than 35 kg/m^2^ [41, 42]. However, SG also has a significant impact on T2DM remission [24, 43, 46]. Prior RCTs have suggested similar outcomes after RYGB and SG with regard to glucose metabolism [15, 44, 47]. The incidence of GERD seems to be more frequent after SG whereas RYGB is considered a therapeutic option in patients with GERD [15, 45, 48]. Nevertheless, the course of GERD after SG is controversial and definite evidence supporting either side does not exist [23, 49, 50]. The current discussion among bariatric surgeons is almost unidirectional and focused on “hard” clinical facts such as weight loss, T2DM remission, and the course of other obesity-related co-morbidities. “Soft” clinical facts such as QoL have gained importance. At the present, there is few literature about patients’ expectation concerning surgical intervention. Data from the Michigan Bariatric Surgery Collaborative on a total of 8.847 patients showed an increased qoL after SG and RYGB [51]. However long-term results of QoL after RYGB and SG are not available. The combined evaluation within this trial of the primary endpoint of EWL after 24 months plus the course of EWL, obesity-related co-morbidities, GERD, morbidity and mortality over 5 years, will lead to further insights of the pros and cons of both procedures. Additionally, the setting of this multi-centre, randomized trial enables a maximum reduction of bias and increases internal and external validity [52].

### Trial status

Recruitment started in November 2013.

## Abbreviations

RYGB: Roux-en-Y gastric bypass
SG: Sleeve gastrectomy
BMI: Body mass index
GERD: Gastroesophageal reflux disease
EWL: Excess weight loss
T2DM: Type 2 diabetes mellitus
QoL: Quality of life
GIQLI: Gastrointestinal quality of life index
GSRS: Gastrointestinal symptom related score
SF-36: Short-Form-36 Health Survey
